# A DJ-1 Based Peptide Attenuates Dopaminergic Degeneration in Mice Models of Parkinson's Disease via Enhancing Nrf2

**DOI:** 10.1371/journal.pone.0127549

**Published:** 2015-05-29

**Authors:** Nirit Lev, Yael Barhum, Tali Ben-Zur, Israel Aharony, Lena Trifonov, Noa Regev, Eldad Melamed, Arie Gruzman, Daniel Offen

**Affiliations:** 1 Felsenstein Medical Research Center, Sackler School of Medicine, Tel-Aviv University, Petach Tikva, Israel; 2 Department of Neurology, Rabin Medical Center, Beilinson Campus, Petach Tikva, Israel; 3 Division of Medicinal Chemistry, Department of Chemistry, Faculty of Exact Sciences, Bar-Ilan University, Ramat-Gan, Israel; Florey Institute of Neuroscience and Mental Health, The University of Melbourne, AUSTRALIA

## Abstract

Drugs currently used for treating Parkinson's disease patients provide symptomatic relief without altering the neurodegenerative process. Our aim was to examine the possibility of using DJ-1 (PARK7), as a novel therapeutic target for Parkinson's disease. We designed a short peptide, named ND-13. This peptide consists of a 13 amino acids segment of the DJ-1-protein attached to 7 amino acids derived from TAT, a cell penetrating protein. We examined the effects of ND-13 using in vitro and in vivo experimental models of Parkinson's disease. We demonstrated that ND-13 protects cultured cells against oxidative and neurotoxic insults, reduced reactive oxygen species accumulation, activated the protective erythroid-2 related factor 2 system and increased cell survival. ND-13 robustly attenuated dopaminergic system dysfunction and in improved the behavioral outcome in the 6-hydroxydopamine mouse model of Parkinson's disease, both in wild type and in DJ-1 knockout mice. Moreover, ND-13 restored dopamine content in the 1-methyl-4-phenyl-1,2,3,6-tetrahydropyridine mouse model. These findings validate DJ-1 as a promising therapeutic target in Parkinson's disease and identify a novel peptide with clinical potential, which may be significant for a broader range of neurological diseases, possibly with an important impact for the neurosciences.

## Introduction

Current treatments for Parkinson’s disease (PD) provide temporary relief of symptoms without altering the disease progression. The development of disease modifying treatments requires profound understanding of the neurodegenerative mechanisms leading to disease progression. Significant advances in the understanding of these mechanisms have been made in the past years with the identification of distinct genetic loci which are associated with pathogenic mutations of PD [1].

Homozygous loss-of-function mutations in DJ-1 (PARK7) result in early-onset familial PD [2–5]. Alterations in DJ-1 levels or isoforms have been documented in brains [6–8], cerebrospinal fluid [9–11] and plasma [12] of sporadic PD patients, mplying a role also in the common sporadic disease. Extensive postmortem studies have provided evidence to support the involvement of oxidative stress in the pathogenesis of PD; these include impaired mitochondrial function, alterations in the antioxidant protective systems and evidence of oxidative damage to lipids, proteins, and DNA [13]. DJ-1 is a ubiquitous redox-responsive cytoprotective protein that protects against oxidative and neurotoxic insults, among them dopamine toxicity [14–20]. DJ-1 has diverse functions, preserving mitochondrial function, regulating redox signaling kinase pathways and acting as a transcriptional regulator affecting anti-oxidant genes and dopamine homeostasis [21–26].

The transcription factor nuclear factor erythroid-2 related factor 2 (Nrf2) is considered the master regulator of redox homeostasis. Nrf2 is a critical inducer of the regulated expression of numerous genes involved in xenobiotic and reactive oxygen species detoxification [27,28]. These genes are collectively termed phase-2 enzymes and share a common promoter enhancer named “antioxidant response element” that is regulated by Nrf2. Among many others, these phase-2 genes encode hemeoxygenase-1 (HO-1), NAD(P)H quinine oxidoreductase-1, GPx, glutathione-reductase, and the catalytic and modulatory subunits of g-glutamyl cysteine ligase [27,28]. In our previous study we demonstrated that DJ-1 plays a role in the disease process in ALS mouse model and the DJ-1 derived peptide, ND-13, enhanced the resistance to glutamate and SIN-1 induced toxicity [29]. In the current study we identify and characterize a DJ-1 derived peptide and tested its potential in animal models of Parkinson's disease.

## Materials and Methods

### Ethics statement

All animal experiments were carried out according to the National Institute of Health (NIH) guidelines and approved by the Ethical Committee of the Tel-Aviv University, Israel. All surgery was performed under subcutaneous injections of a mixture of ketamine (100mg/kg) and xylazine (8mg/kg) anesthesia, and all efforts were made to minimize suffering. Animals were sacrificed by CO_2_ exposure.

### DJ-1 based peptide: ND-13

ND-13, a 20 amino-acids (aa) peptide, is composed of a 13 aa DJ-1-based peptide, attached to a TAT-derived 7 aa sequence, that serves as a cell penetrating peptide (CPP). TAT-derived CPP’s have previously been shown to facilitate the delivery into the central nervous system [30–33]. The ND-13 sequence is YGRKKRRKGAEEMETVIPVD [29].

### Cell cultures, treatments, and evaluation of oxidative stress and cell viability

Human neuroblastoma cell-line, SH-SY5Y and rat adrenal medullary cell-line, PC-12, were obtained from ATCC (Rockville, USA). Primary neuronal cultures were prepared from newborn mice (24–48 hr), as previously reported [34]. Cells were exposed to increasing doses of H_2_O_2,_ 6-OHDA or dopamine (Sigma, USA). The ND-13 peptide, or control peptides (short peptides based on DJ-1 attached to TAT) were synthesized in Peptide 2.0 (USA), BioSight (Israel), or ChinaPeptides (China). The peptides were freshly dissolved in phosphate buffered saline (Biological-Industries, Beit Haemek, Israel) for in vitro studies, or in saline for in vivo studies. Peptides were added to the cells in serum free medium one hour before exposure to oxidative or toxic insults. The medium was changed and cells were washed before toxic insults were applied. All experiments were executed in serum free medium. Each experiment was repeated at least 3 times.

Cell toxicity was measured by Alamar blue assay, a redox indicator that changes color in response to metabolic activity and by lactate dehydrogenase (LDH) cytotoxicity detection kit (Clontech-Laboratories, USA).

Cell proliferation was assessed by the 5-bromo-2′-deoxyuridine (BrdU) assay (Millipore, USA) according to the manufacturer’s protocol; results were read at 450/550 wavelength using an ELISA reader (Powerwave X, Biotek-Instruments, USA).

After exposure to increasing concentrations of oxidative insults, the generation of intracellular reactive oxygen species was measured using H_2_DCFDA (Sigma). DCF fluorescence was assessed by FLUOstar spectrofluorometer microplate reader at 520nm.

### RNA extraction, cDNA synthesis and real time Polymerase Chain Reaction (PCR)

RNA extraction, cDNA synthesis and real time PCR were done as previously reported [23]. The primers used are Nrf2 forward ACA TCC AGA CAG ACA CCA GTG G, Backward TGT GGG CAA CCT GGG AGT AG, HO-1 forward GCC TGC TAG CCT GGT GCA AG backward AGC GGT GCT TGG GAT GAG CTA, NQO-1 forward CAT TCT GAA AGG CTG GTT TGA, backward CTA GCT TTG ATC TGG TTG TCA G, GCLC forward ACA AGC ACC CCC GCT TCG GT backward CTC CAG GCC TCT CTC CC

### Protein extraction and Western blotting

Whole cell protein extraction and Western blotting were performed as previously reported [23]. Membranes were probed with anti HO-1 (1:500), and anti beta-actin (1:2000). Visualization and analysis of band intensities were performed using the Odyssey system (LICOR, Lincoln, NE, USA).

### Nrf2 activity kit

Cytoplasm and nucleus proteins extracted by optimized Nuclear Extract kit (Active Motif, CA). Nuclear Nrf2 activity, measuring the ARE oligonucleotide DNA binding activity of the Nrf2, was quantified using the TransAM Nrf2 activity kit (Active Motif, CA), according to the manufacturer’s instructions.

### Wild Type and DJ-1 knockout mice

c57/bl6 male mice were purchased from Harlan, Israel at the age of 6–8 weeks (22–28 gr). Transgenic DJ-1 knockout mice were purchased from the Jackson Laboratory (Bar Harbor, ME, USA). Animals were housed at a temperature (22 ± 1°C), humidity (30%), and in a light-controlled environment (12-h light/ dark cycle) with ad libitum access to food and water. Animals were acclimatized for 1 week prior to experimentation then randomly divided into the experimental groups, n = 10.

### In vivo fluorescence monitoring

To monitor the ability of ND-13 to penetrate the blood-brain barrier in vivo, the CellVizio system (Manua-Kea Technologies) enabling the detection of fluorescence in blood vessels and its accumulation in the brain parenchyma, was used. Mice were injected IV with 50mg/kg FITC-Albumin (Sigma-Aldrich) in order to verify the integrity of the blood brain barrier. 10 minutes post injection, mice were anesthesized using subcutaneous injections of Ketamine (100mg/kg) and Xylazine (8mg/kg) mixture. Next, the mice were placed in a stereotaxic device, and a 0.3mm fluorescence detection probe was inserted to the central caudate-putamen using the following coordinates: +0.5 mm anterior, 2.0 mm lateral to bregma, and 2.5 mm deep to the skull surface. Fluoresence was monitored every 5 minutes using a 488nm excitation laser. 20 minutes after record start point, the tested peptides were injected subcutaneously and brain fluorescence was monitored for another 30 minutes. Pictures were taken at the same exposure and length conditions.

### 6-OHDA hemiparkinsonian mice model

Eight-week-old male C57BL/6 or DJ-1 knockout mice were used for 6-OHDA hemiparkinsonism model experiments. Mice received a unilateral, right intrastriatal injection of 4 μg 6-OHDA **(**Sigma-Aldrich) using a stereotaxic surgical procedure. Injections were targeted to the central caudate-putamen using the following coordinates: +0.5 mm anterior, 2.0 mm lateral to bregma, and 2.5 mm deep to the skull surface. In the intracerebroventricular treatment, 6-OHDA (2mg/ml), ND-13 (0.2mg/ml) or vehicle (saline), were administered in a volume of 2.0μl,0.5 μl/min.

ND-13 or vehicle were administered systemically by intravenous or subcutaneous injections before or after intrastriatal 6-OHDA lesioning. Intravenous ND-13 was administered (1.5mg/Kg) 4 hours before 6-OHDA lesioning. subcutaneous administration of ND-13 (3 mg/Kg) was 6 hours before 6-OHDA lesioning and one hour after lesioning. Each study was repeated at least twice with 12–15 mice per group.

### Behavioral testing: amphetamine-induced rotation asymmetry

Mice were tested for amphetamine-induced turning behavior 14 and 28 days after the 6-OHDA injection. Motor behavior was recorded by video tracking systems, EthoVision XT (Noldus) for 60 minutes after injection of amphetamine (2.5 mg/kg s.c.). The net rotation asymmetry score for each test was calculated by subtracting contralateral turns from the turns ipsilateral to the lesion.

### 1-methyl-4-phenyl-1,2,3,6-tetrahydropyridine (MPTP) mice model

Adult male 8-week-old C57/bl and DJ-1 knockout mice (25 gr) were injected intraperitoneally daily, for five consecutive days, with MPTP, at a dose of 25 mg/kg. Doses of 3 or 20 mg/kg of ND-13, or control peptides in 100μl saline were administered subcutaneously, 10 minutes before the MPTP injections. As control peptides, we used two short DJ-1-related peptides (6aa and 8aa), attached to TAT, or the ND-13 without TAT moiety (ND-13C). After two weeks, the mice were sacrificed and the dopamine content and immunochitochemistry for tyrosine hydroxylase were examined, as elaborated below. The experiment was repeated 3 times, n = 6–10 per group.

### Analysis of dopamine content

Each brain hemisphere was subjected to analysis by high performance liquid chromatography (HPLC). Dopamine, 3,4-dihydroxyphenylacetic acid and homovanillic acid concentrations in tissue samples were determined by HPLC with electrochemical detection.

### Tissue processing and immunohistochemistry

Fourteen days after MPTP, treatment or one month after 6-OHDA lesioning, mice were perfused. Brains were removed and fixed. 10-μm-thick cryosections on slides were incubated with mouse anti-TH antibodies (1:500, Sigma). Subsequently, we used the Vectastain Elite ABC kit and 3, 3'-diaminobenzidine peroxidase substrate kit (Vector-laboratories, CA, USA) following the manufacturer’s instructions.

### Statistical analysis

Comparisons of two groups were conducted using a 2- tailed Student’s t test. Statistical analyses for three or more groups were performed using analysis of variance followed by least-significant difference post hoc comparison. Differences among groups were considered significant if the probability of error (p) was less than 5%.

## Results

### ND-13 attenuates neurotoxic and oxidative insults

We used a thorough bio-informatic survey and knowledge gained in previous studies to locate conserved areas in the DJ-1 protein. We then designed a series of short peptides (up to 20 amino acids) and using an in vitro cellular platform based on SH-SY5Y cells. We chose the peptide that most effectively protected against oxidative and toxic insults. In order to enable cell penetration, we attached the peptide to diverse CPP. The most promising peptide we analyzed is a short peptide composed of 13 amino-acids derived from DJ-1, attached to a 7 amino-acids sequence derived from TAT that served as the CPP moiety. The combined peptide was named ND-13. In order to verify that ND-13 penetrates the cells, PC-12 cells exposed to ND-13, vehicle or the TAT-derived CPP alone were immunostained with antibodies against TAT. Immunocytochemical staining demonstrated that ND-13 indeed penetrates the cells (not shown).

**Fig 1:**
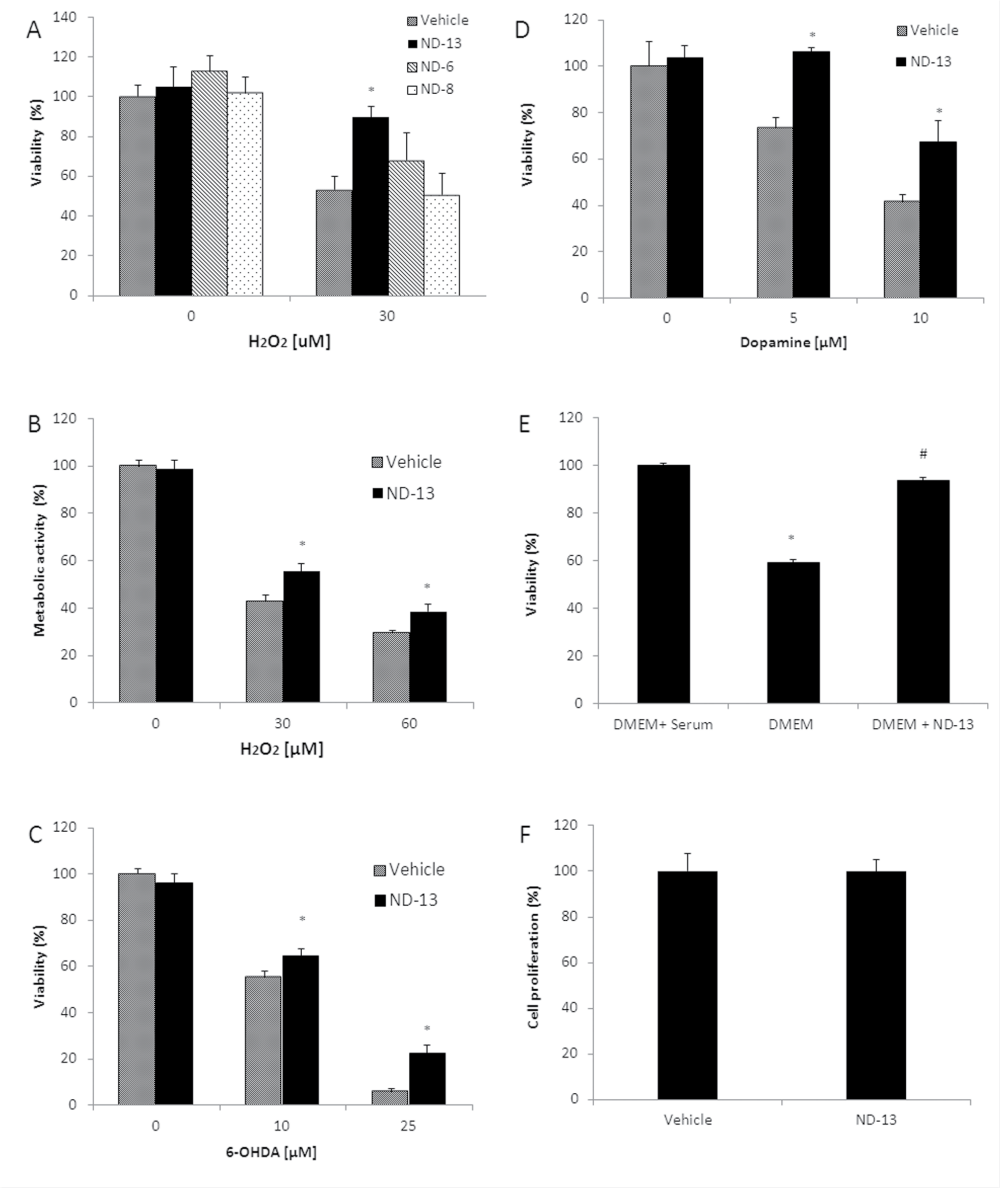
ND-13 attenuates toxicity of trophic factors depletion, oxidative insults and neurotoxins. SH-SY5Y, human neuroblastoma cells, were exposed to increasing doses of hydrogen peroxide (H_2_O_2_, 0–60 μM), 6-OHDA (0–25 μM) or dopamine (0–10 μM) for 24 hours. ND-13 (4 μM) or vehicle were applied 1 hour before the toxic insults. (A) ND-13 attenuated H_2_O_2_-induced cell death, measured by LDH cytotoxicity assay and (B) restored H_2_O_2_-induced metabolic changes, measured by Alamar blue. As controls we used cells treated with vehicle (phosphate buffered saline) or short peptides (ND-6 and ND-8) with sequences similar to ND-13, attached to the same cell penetrating peptide as ND-13. (C) ND-13 attenuated 6-OHDA and (D) against dopamine toxicity, as measured by the LDH cytotoxicity assay. (E) ND-13 preserved viability of human neuroblastoma cells SH-SY5Y exposed to trophic factors depletion (done by serum deprivation for 48 hours, * p<0.05 as compared to cell culture with serum, # p<0.05 as compared to cell culture without serum or ND-13). (F) ND-13 does not induce cell proliferation. Human neuroblastoma cells (SH-SY5Y) were exposed to 4 μM ND-13 or vehicle for 24 hours. Cell proliferation was quantified by BrdU assay. No significant differences were observed between ND-13 treated cells and vehicle treated cells. Data are presented as means ± S.D. * p<0.05 as compared to cells exposed to vehicle and the same toxic dose. The experiments were repeated 3 times in triplicates.

We exposed cells treated with ND-13 or vehicle to oxidative or neurotoxic insults and measured several cellular injury parameters: cell death was quantified by measuring LDH release into the medium, and metabolic damage was quantified by Alamar blue assay. ND-13 increased cell viability and reduced metabolic damage after exposure to H_2_O_2_ (Fig 1A and 1B). ND-13 also significantly attenuated neurotoxic insults induced by exposure to 6-OHDA and dopamine (Fig 1C and 1D), and serum deprivation (Fig 1E). Using a BRDU incorporation kit, we found that ND-13 did not increase cell proliferation (Fig 1F).

**Fig 2:**
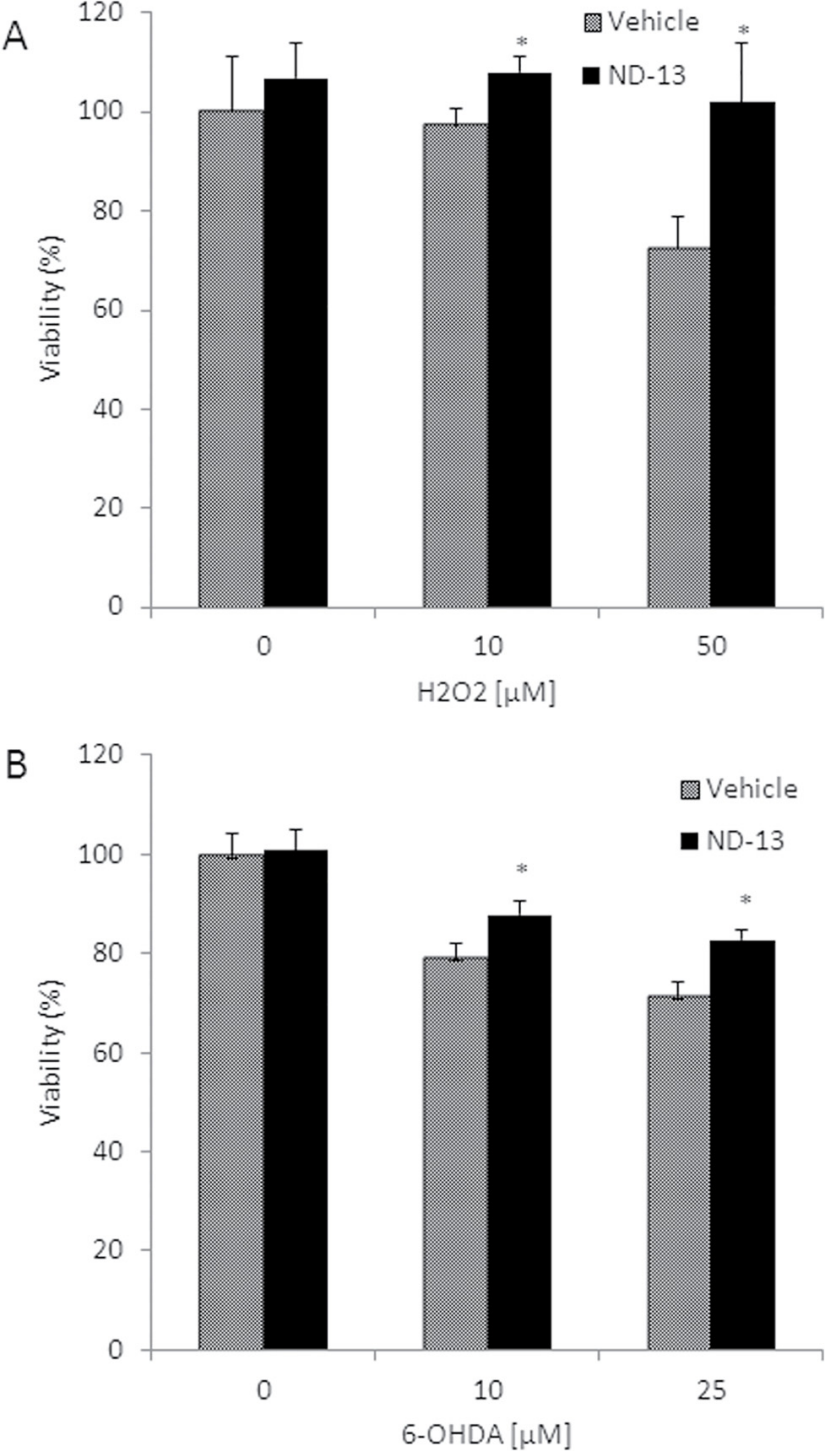
ND-13 peptide attenuated toxic-induced cell death in primary neuronal cultures. Primary neuronal cultures were prepared from c57/bl6 mice brains, as elaborated in the methods section. Cells were exposed to increasing doses of hydrogen peroxide (H2O2, 0–50 μM) (A), 6-OHDA (0–25 μM) (B) for 24 hours. ND-13 (4 μM) or vehicle were applied 1 hour before the toxic insults. (A) ND-13 prevented H2O2-induced cell death and (B) 6-OHDA-induced cell death, measured by LDH cytotoxicity assay. * P<0.05 as compared to control cells exposed to vehicle. The experiments were repeated 3 times in triplicates.

ND-13's ability to attenuate oxidative and neurotoxic insults was also examined on primary neuronal cultures obtained from C57/bl6 mice brains. Similar to the results in cell lines, ND-13 significantly prevented cell death induced by H_2_O_2_ and 6-OHDA toxicity in primary neuronal cultures (Fig 2).

### ND-13 reduced intracellular oxidative stress, activating the Nrf2 system

**Fig 3:**
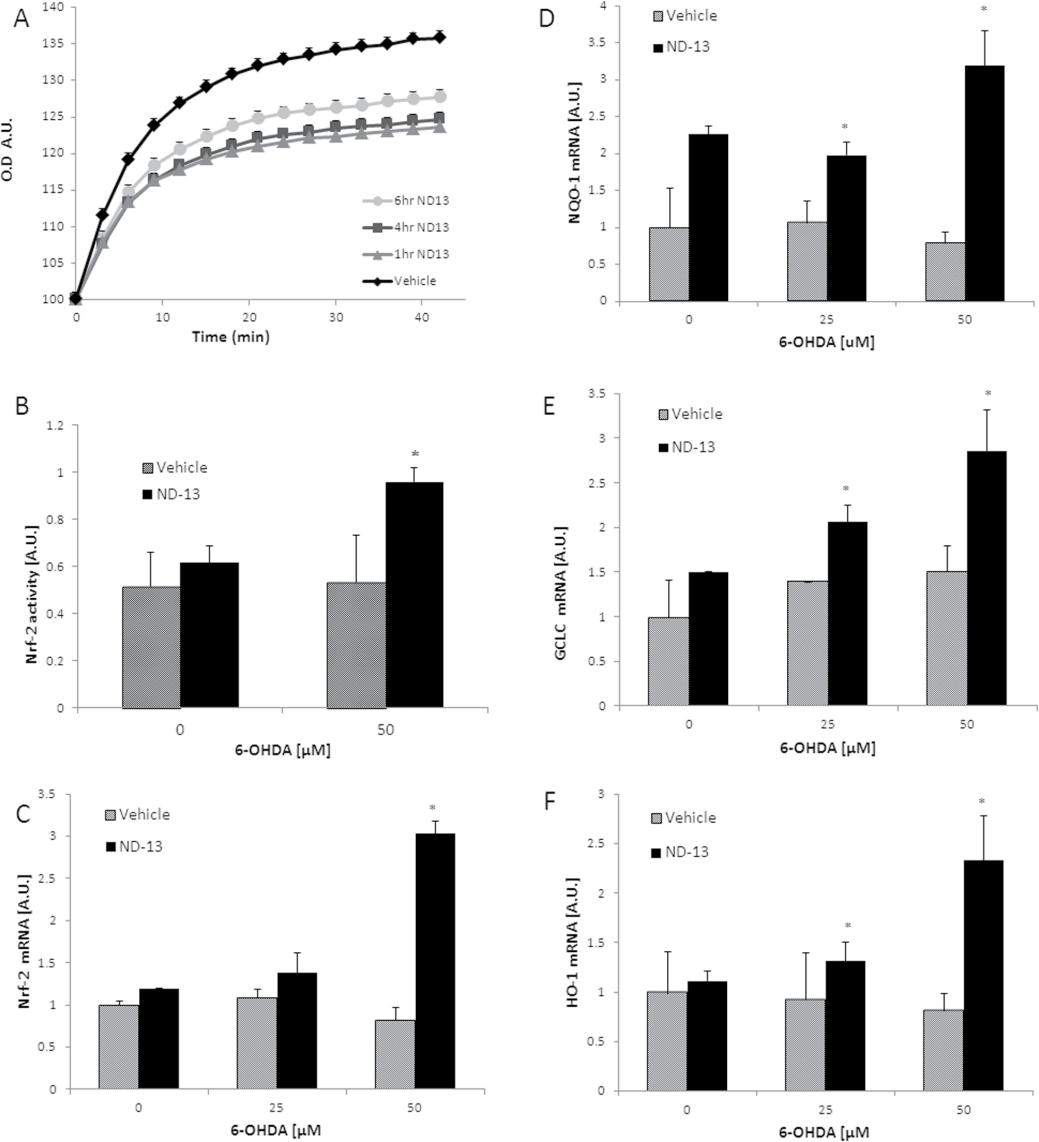
ND-13 reduced the accumulation of intracellular reactive oxygen species and activated the Nrf2 anti-oxidant system. (A) Exposure to hydrogen peroxide resulted in intracellular accumulation of reactive oxygen species, as quantified by the DCF assay. Pretreatment with ND-13 resulted in significantly decreased accumulation of reactive oxygen species. The time dependent effect was examined by increasing the time between ND-13 treatment and the oxidative insult. ND-13 showed significant effects when applied up to 6 hours before hydrogen peroxide exposure. Cells were washed before toxin application. (B) ND-13 induced nuclear translocation of Nrf-2, as quantified by cell fractionation and the Nrf2 activity kit (TransAM) on nuclear extracts. Results are shown as averages ± SD. * p<0.05. (C-F) Pretreatment with ND-13 induced early and increased expression of Nrf-2-dependent mRNA of antioxidant genes when cells were exposed to the neurotoxin 6-OHDA. (C) Quantitative real time PCR analysis of the mRNA levels of Nrf2, (D) NAD(P)H quinine oxidoreductase-1, GCLC (E) and HO-1 (F) levels in PC-12 cells, 2 hours after exposure to increasing doses of 6-OHDA (0–50 μM). Real time PCR was done in triplicate, by the ddCT method, with glyceraldehyde 3-phosphate dehydrogenase used as a housekeeping gene. Experiments were repeated 3 times. Results are shown as averages ±SD. * p<0.05. (G, H) Western blots of HO-1 protein levels in cells exposed to increasing doses of 6-OHDA (0–50 μM) for 8 hours. ND-13 pretreated cells showed increased HO-1 protein levels (normalized versus beta-actin levels) after exposure to 6-OHDA.

Accumulating evidence indicates that one of the mechanisms through which DJ-1 exerts antioxidative stress response [14–20], is through the activation of Nrf2, the master regulator of redox homeostasis. Therefore, we first examined ND-13’s ability to reduced oxidative damage. Exposure of human neuroblastoma cells SH-SY5Y to hydrogen peroxide resulted in the accumulation of intracellular reactive oxygen species with time, as was quantified by the intracellular 2,7-dichlorofluorescin (DCF) method (Fig 3A). Pretreatment with ND-13 (20 μM), up to 6 hours before toxin exposure, significantly attenuated intracellular reactive oxygen species accumulation (Fig 3A).

**Fig 4:**
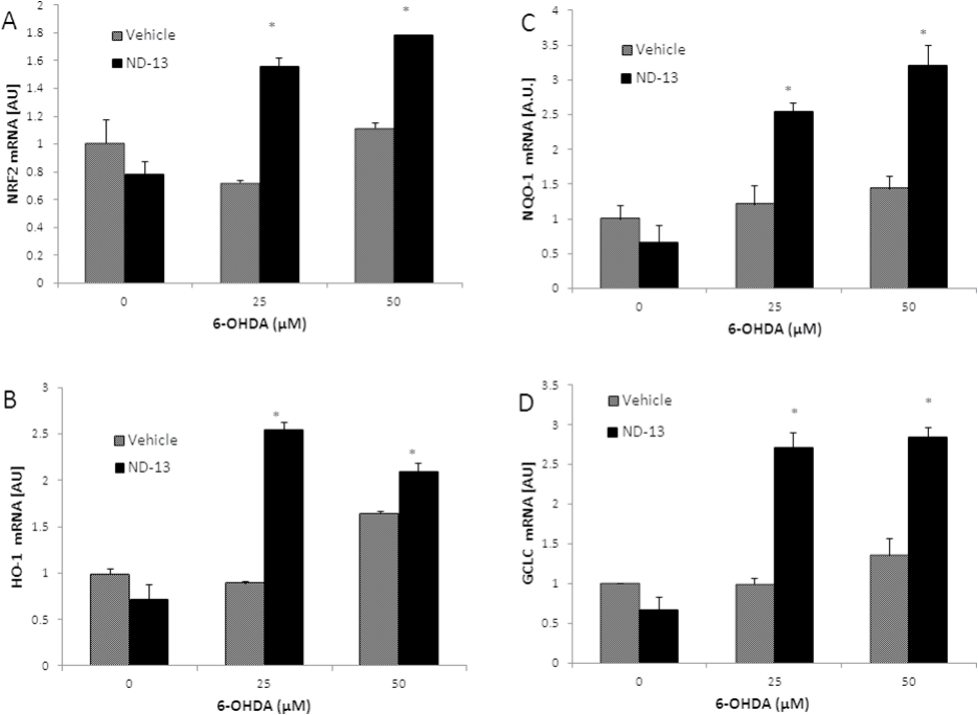
ND-13 increased activation of the Nrf2 after exposure to 6-OHDA. (A) Quantitative real time PCR analysis of the mRNA levels of Nrf2, (B) HO-1, (C) NQO-1 and (D) GCLC levels of PC-12 cells, 5 hours after exposure to increasing doses of 6-OHDA (0–50 μM). Real time PCR was done in triplicate. GAPDH was used as a housekeeping gene. Real time PCR was done by the ddCT method. Experiments were repeated 3 times. Results are shown as averages ±SD. * p<0.05.

To examine the involvement of Nrf2 in ND-13’s activity, we measured the nuclear translocation of Nrf2 with and without ND-13 treatment. Using cell fractionation and a quantitative Nrf2 activity kit (TransAM), we found that ND-13 exposure by itself did not significantly cause translocation of Nrf2 into the nucleus (Fig 3B). However, when cells pre-treated with ND-13 were exposed to an oxidative insult such as 6-OHDA, a significantly increased nuclear translocation and Nrf2 binding to the antioxidant response element promoter were detected (Fig 3B). Next, we examined the expression of Nrf2-regulated antioxidant genes as well as the mRNA for Nrf2 in ND-13 versus vehicle-treated cells, with and without exposure to oxidative insults. Real-time PCR analysis revealed early and significant increases in the expression of Nrf2-regulated antioxidant genes HO1, NAD(P)H:quinone oxidoreductase 1 and glutamate cysteine ligase in ND-13-treated cells exposed to oxidative insults (Fig 3C–3F). Two hours after exposure to the toxic insult induced by 6-OHDA, a significantly increased mRNA expression of Nrf2 genes was observed in cells pre-treated with ND-13, while no significant upregulation of these genes’ expression was observed in cells treated with vehicle (Fig 3C–3F). Five hours after exposure to 6-OHDA, cells treated with vehicle did show some upregulation of Nrf2 genes; but those cells which were pretreated with ND-13 showed significantly higher mRNA levels of these antioxidant genes (Fig 4).

### ND-13 effects on an in vivo 6-OHDA hemiparkinsonian mice model

**Fig 5:**
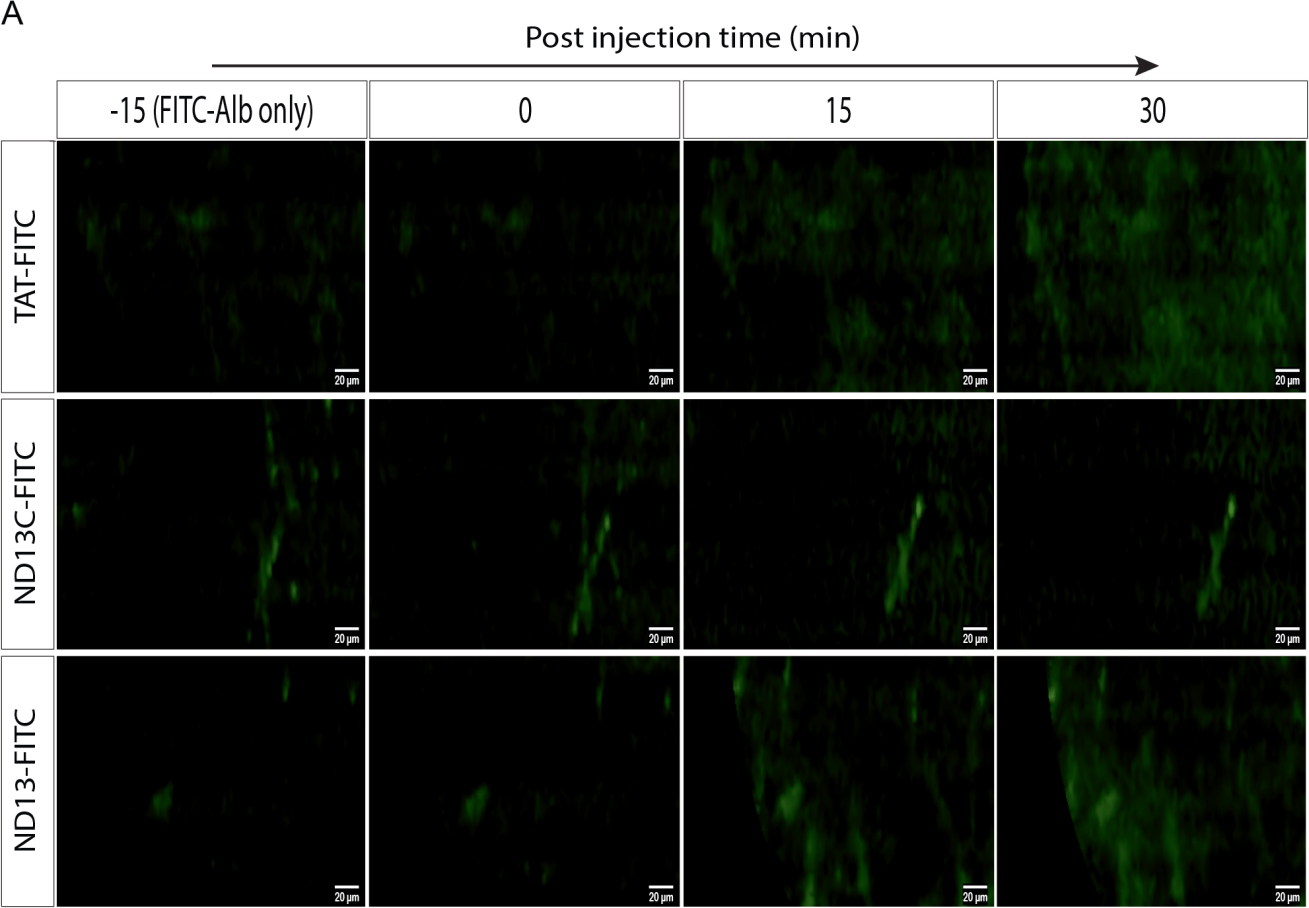
ND-13 blood-brain-barrier penetration. C57/bl6 mice were injected with FITC-Albumin (FITC-Alb) to visualize intact blood vessels. FITC conjugated TAT peptide (FITC-TAT), FITC conjugated ND-13 lacking the TAT sequence (ND-13C-FITC) or FITC conjugated full length ND-13 (ND-13-FITC) were injected subcutaneous (SC) and monitored by CellVizio system for additional 30 min. Time dependent accumulation of the fluorescent signal in the brain parenchyma around the blood vessels was observed when mice were administrated with either TAT-FITC or ND-13-FITC but not with ND-13C, indicating that the TAT-derived cell penetrating peptide enables ND-13 to penetrate the BBB.

In order to evaluate ND-13's ability to penetrate the blood-brain barrier in-vivo, we monitored in the brain parenchyma, the accumulation of ND-13, labeled with a fluorescein isothiocyanate (FITC) tag at the C-terminal. Following ND-13C-FITC injection, the signal remained in the blood vessels while 15 min after TAT-FITC and ND-13-FITC injection we observed diffused fluorescence in the brain parenchyma indicating blood brain barrier penetration (Fig 5).

**Fig 6:**
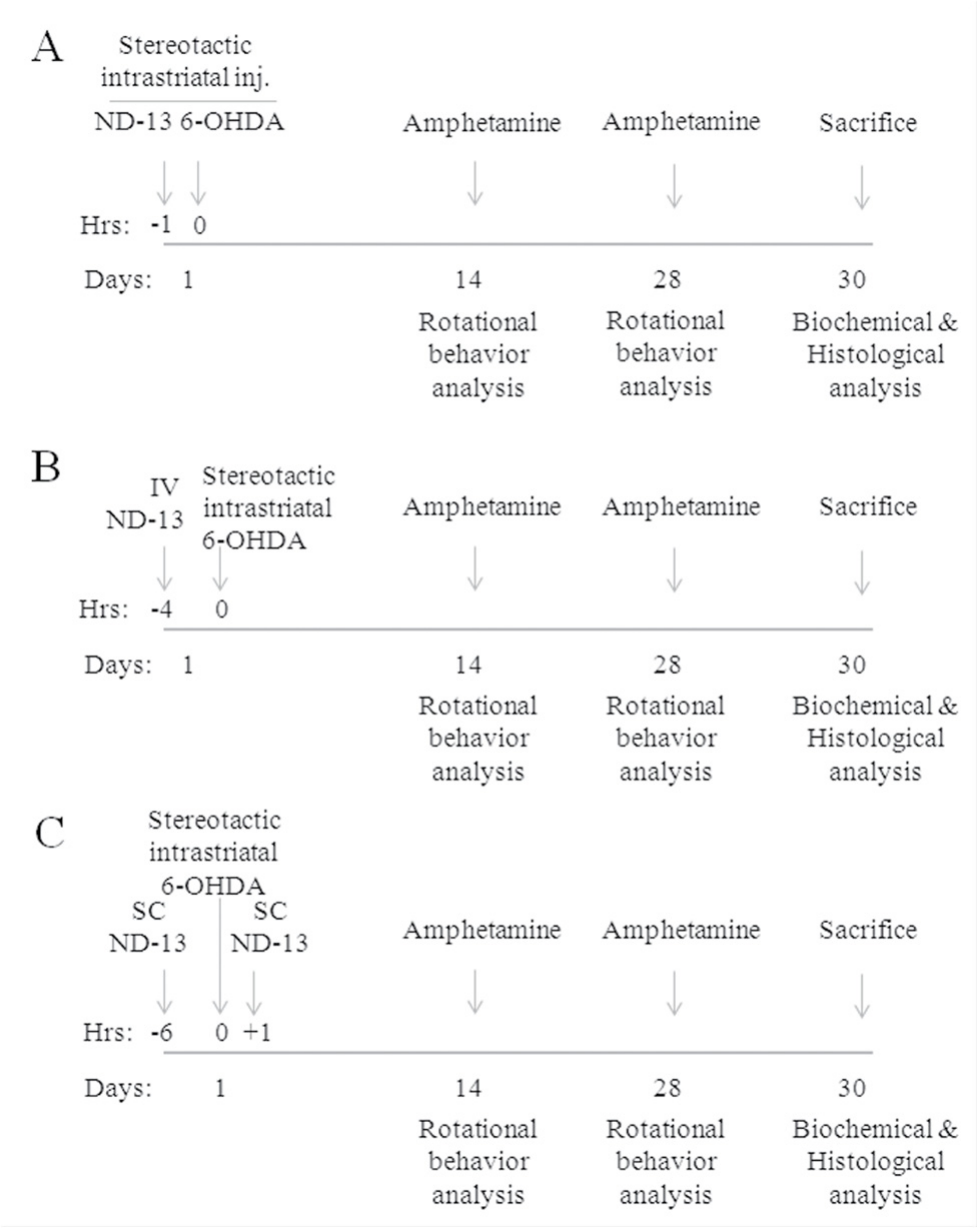
In vivo experimental designs. Experimental designs of the in vivo 6-OHDA hemiparkinsonian mice experiments are shown. (A) Design of the stereotactic intracerebral ND-13 versus vehicle experiment. (B) Design of the intravenous (IV) administration experiment. (C) Design of the subcutaneous (SC) experiment.

**Fig 7:**
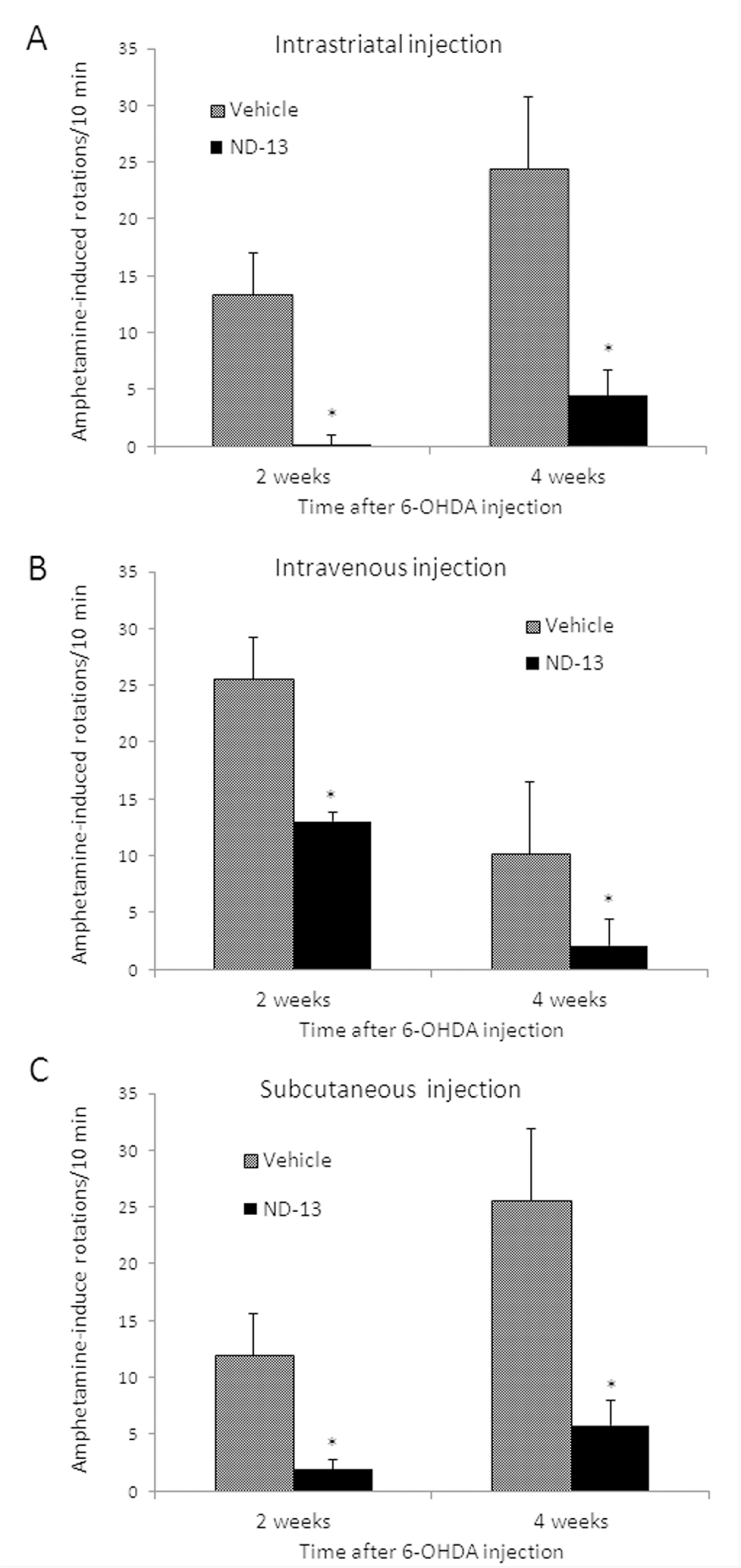
ND-13 attenuates 6-OHDA toxicity in c57/bl6 mice. A 6-OHDA hemiparkinsonian mice model was induced by right striatal stereotactic 6-OHDA injection as elaborated in the methods section. Amphetamine-induced rotational behavior was used to quantify the damage to the dopaminergic neurons. (A) Intracerebral injection of ND-13 (0.4 μg in 2 μl saline) markedly reduced the rotational behavior induced by amphetamine injection, 2 and 4 weeks after 6-OHDA striatal lesioning, as compared to vehicle-treated 6-OHDA mice. (B) Intravenous administration of ND-13 (1.5 mg/Kg dissolved in 200μl saline, 4 hours before 6-OHDA) significantly reduced the rotational behavior induced by amphetamine injection, 2 and 4 weeks after 6-OHDA striatal lesioning. (C) Subcutaneous administration of ND-13 (3 mg/Kg dissolved in 100μl saline, 6 hours before 6-OHDA and one hour after lesioning) significantly reduced the rotational behavior induced by amphetamine injection, 2 and 4 weeks after 6-OHDA striatal lesioning. Results are shown as averages ± SD. * p<0.05.

Using a stereotaxic surgical procedure, C57/bl6 or DJ-1 knockout transgenic mice were striatally lesioned by 6-OHDA. The mice were treated before or after striatal 6-OHDA lesioning with ND-13 or vehicle, administered intracerebrally, intravenously or subcutaneously (see experimental designs in Fig 6). Dopaminergic cell depletion induced by 6-OHDA damage increases the asymmetry between the brain hemispheres and induces rotational behavior after amphetamine injection. Intracerebral administration of ND-13 significantly reduced amphetamine-induced rotations, as quantified 2 weeks and 4 weeks after the lesioning and compared to vehicle-treated 6-OHDA mice (p<0.05) (Fig 7A).

Next, the effect of systemic administration of ND-13 was examined by an intreavenous (IV) injection of 1.5mg/kg ND-13, 4 hours before the intracerebral stereotactic 6-OHDA lesioning (Fig 7B). Significant behavioral amelioration was noted, as evidenced by a significant decrease in amphetamine-induced rotations in the ND-13 treated mice, 2-weeks and 4-weeks after 6-OHDA lesioning, as compared to vehicle-treated mice (Fig 7B).

Subsequently, we examined the effect of subcutaneous administration of ND-13 (3mg/kg), 6 hours before and 1 hour after 6-OHDA lesioning (Fig 7C). It was found that SC administration significantly reduced amphetamine-induced rotational behavior 2 and 4 weeks after 6-OHDA lesioning (Fig 7C)

**Fig 8:**
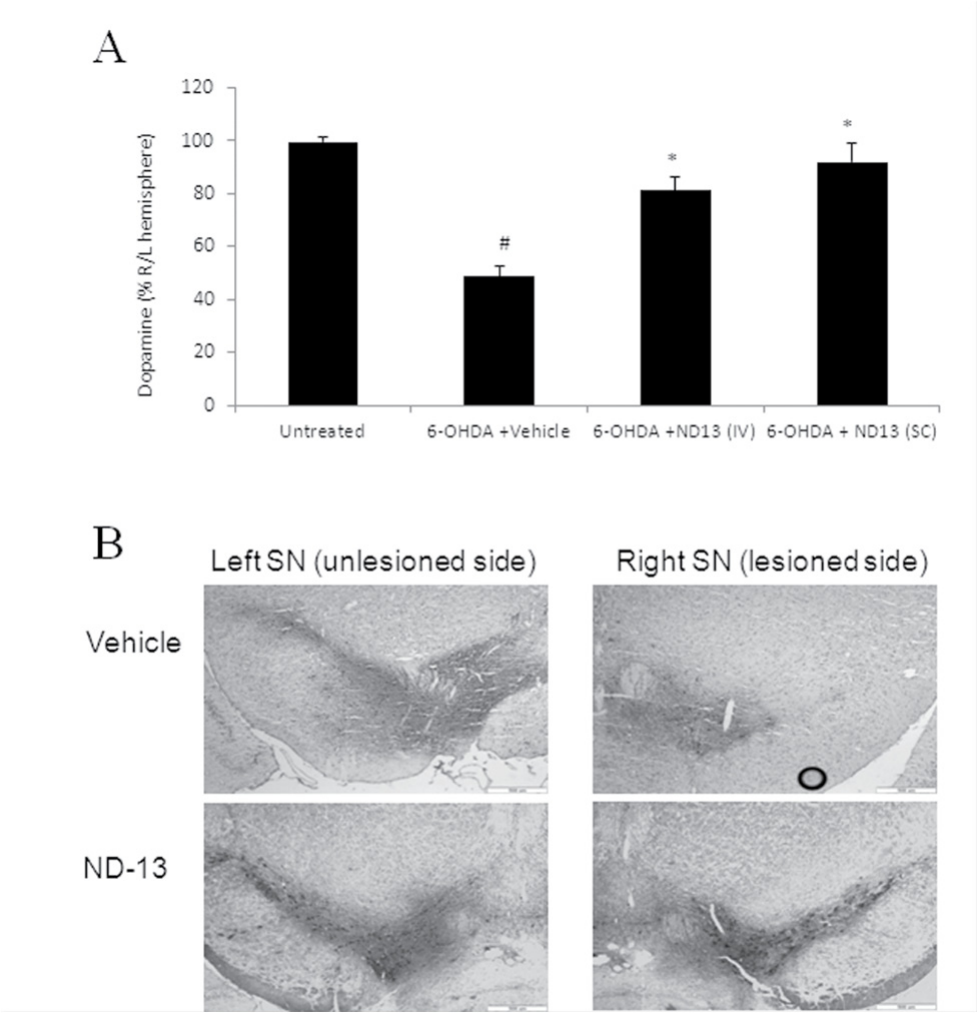
ND-13 restores dopamine levels and tyrosine hydroxylase in 6-OHDA hemiparkinsonian mice. (A) Dopamine levels in brains of naïve (control) mice or mice lesioned by 6-OHDA were measured by HPLC. Dopamine level in the right (6-OHDA lesioned) hemisphere is presented as percentage of the normal left hemisphere. IV injection of the ND-13 (1.5 mg/Kg dissolved in 200 μl, 4 hours before 6-OHDA) or SC injection of ND-13 (3 mg/Kg dissolved in 100 μl saline, 6 hours before and 1 hour after 6-OHDA lesioning) restored dopamine levels as compared to vehicle treated 6-OHDA mice (# p<0.05, as compared to naïve mice, * p<0.05, as compared to vehicle-treated 6-OHDA mice). (B) IV administration of ND-13 (1.5 mg/Kg dissolved in 200μl saline, 4 hours before 6-OHDA lesioning) markedly reduced the loss of tyrosine hydroxylase (TH) staining in the 6-OHDA-lesioned (right) substantia nigra in wild type c57/bl6 mice, as compared to vehicle-treated 6-OHDA mice. TH staining in the left (unlesioned) substantia nigra did not show significant differences.

One month after 6-OHDA lesioning, the mice were sacrificed and hemispheric dopamine content was quantified in each hemisphere by HPLC. ND-13 treated 6-OHDA mice demonstrated a significant restoration of dopamine levels in the lesioned hemisphere, as compared to vehicle-treated mice (Fig 8A). The preservation of tyrosine hydroxylase phenotype in ND-13-treated mice was also confirmed by the immunohistological staining of substantia nigra slices with anti-tyrosine hydroxylase antibodies, demonstrating the degree of damage to the dopaminergic nerve cells (Fig 8B).

### ND-13 preserves dopaminergic system in DJ-1 knockout mice

**Fig 9:**
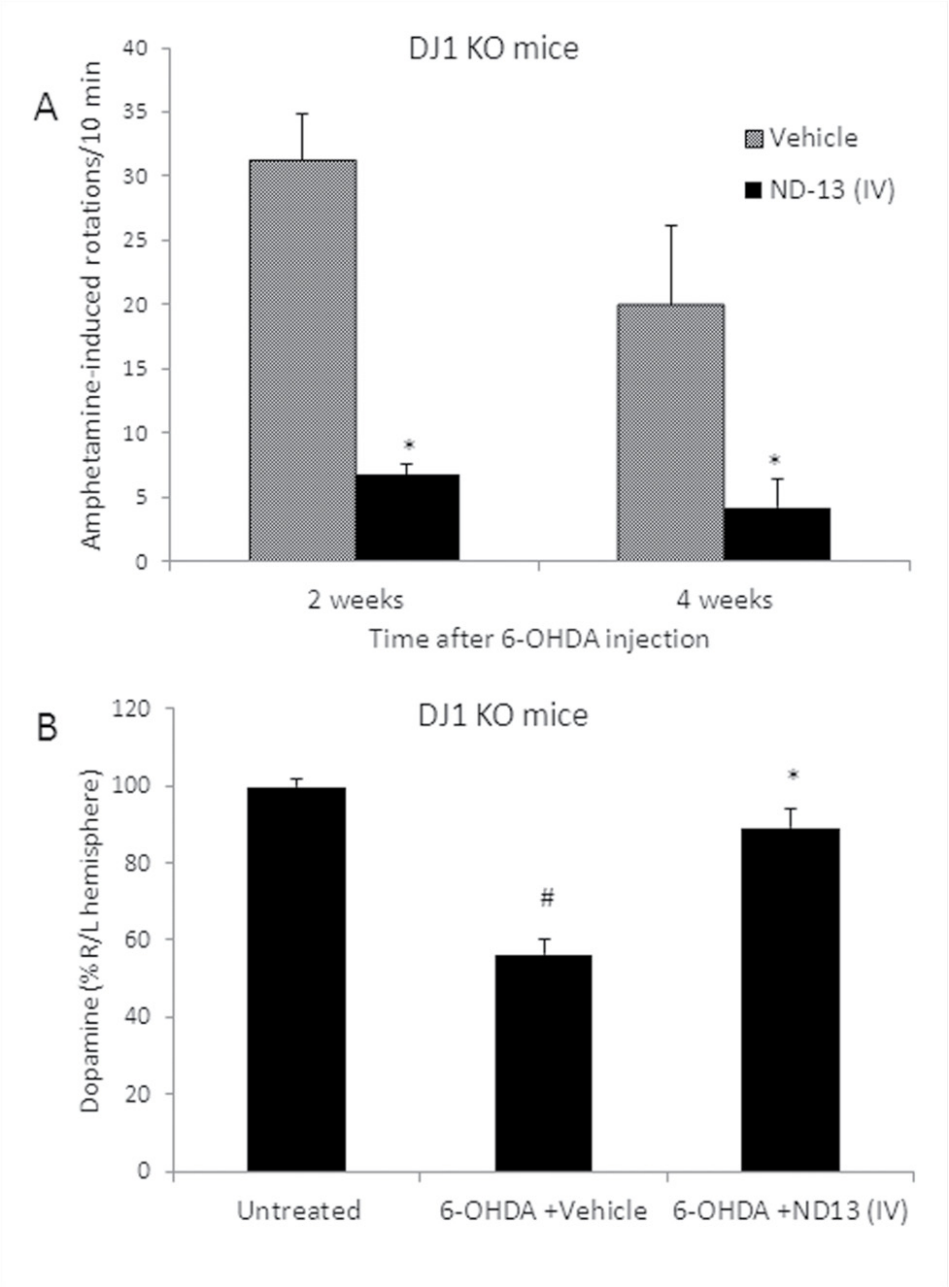
ND-13 administration attenuates 6-OHDA toxicity in DJ-1 knockout mice. (A) IV administration of ND-13 (1.5 mg/Kg dissolved in 200μl saline, 4 hours before 6-OHDA lesioning) significantly reduced the rotational behavior induced by amphetamine injection, 2 and 4 weeks after 6-OHDA striatal lesioning in 6-OHDA hemiparkinsonian transgenic DJ-1 knockout mice. Results are shown as averages ± SD. (# p<0.05, as compared to naïve mice, * p<0.05, as compared to vehicle-treated 6-OHDA mice). (B) IV administration of ND-13 restores dopamine levels, as measured by HPLC in 6-OHDA lesioned DJ-1 knockout mice. Dopamine levels were measured in each cerebral hemisphere of naïve DJ-1 knockout mice (control), or mice lesioned by 6-OHDA treated by ND-13 or vehicle. The dopamine level in the right (6-OHDA lesioned) hemisphere is presented as a percentage of the normal left hemisphere. IV administration of ND-13 (1.5 mg/Kg dissolved in 200 μl, 4 hours before 6-OHDA) statistically restored dopamine levels, as compared to vehicle-treated 6-OHDA mice. Results are shown as averages ± SD. * p<0.05 as compared to vehicle-treated 6-OHDA DJ-1 knockout mice.

Studies on transgenic DJ-1 knockout mice found that these mice are more sensitive to oxidative stress and neurotoxicity [14,15,17]. We examined whether ND-13 protects DJ-1 knockout mice against oxidative stress and neurotoxicity. ND-13 was given intravenously 4 hours before stereotactic intrastriatal injection of 6-OHDA (experimental design shown in Fig 6B). Indeed, we observed 6-OHDA lesioning caused a marked impairment of the dopaminergic system in DJ-1 knockout mice as compared to WT mice. However, similarly to wild type mice, ND-13 significantly attenuated 6-OHDA toxicity in DJ-1 knockout mice. As shown in Fig 9, ND-13-treated DJ-1 knockout mice demonstrated preserved behavior (Fig 9A) and brain dopamine content (Fig 9B) as compared to vehicle treated mice.

### ND-13 attenuates MPTP toxicity in vivo

**Fig 10:**
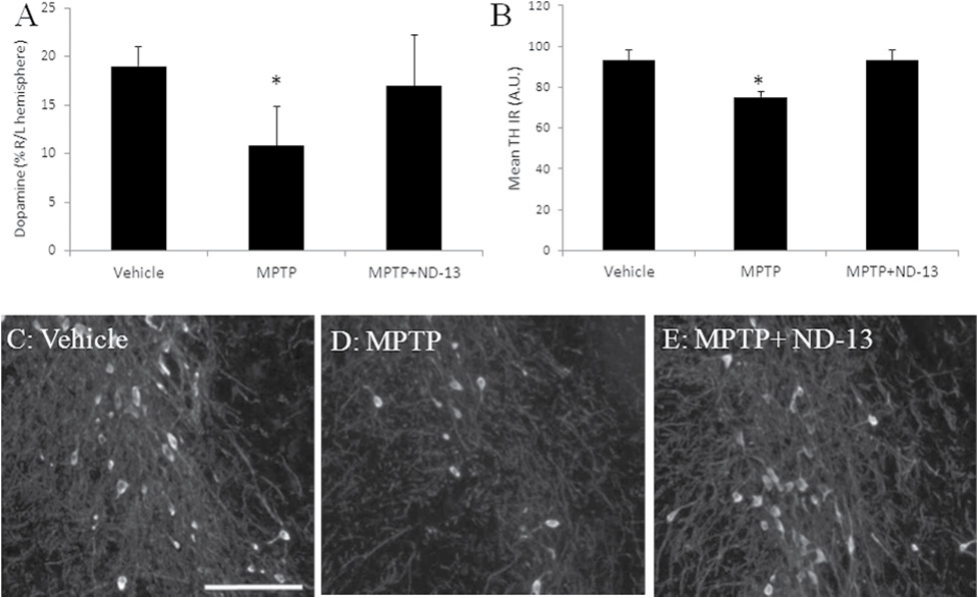
Subcutaneous ND-13 administration preserves dopamine levels in MPTP treated mice. (A) Systemic MPTP injection (25mg/kg) for 5 consecutive days reduced dopamine levels in the brain by selectivetoxicity to dopaminergic cells. Subcutaneous injection of ND-13 (20mg/Kg daily) restored dopamine levels, as measured by HPLC (* p<0.05). (B) Mean intensity of tyrosine hydroxylase immune reactivity area measured in the substantia nigra. Immunohistochemistry of the substantia nigra for tyrosine hydroxylase staining in vehicle-treated mice (C), MPTP-treated mice (D), and ND-13-treated MPTP mice (E). Reduced TH labeling is shown in vehicle-treated MPTP substantia nigra. ND-13 treatment partially restores tyrosine hydroxylase staining. Scale bar = 100 μm.

C57/bl6 mice were injected daily, for five consecutive days, intraperitoneally with MPTP-HCl, (25mg/kg), and ND-13 (20mg/kg), or vehicle subcutaneously, daily in parallel to the MPTP. After two-weeks, the mice were sacrificed and brain dopamine content was measured by HPLC. We observed that subacute injections of MPTP resulted in a decrease in the hemispheric dopamine content (Fig 10A). However, ND-13 significantly restored the brain's dopamine content in the MPTP-injected mice (Fig 10A). The decrease in mean tyrosine hydroxylase immune reactivity area (TH IR) measured in the substantia nigra of MPTP-treated mice, was attenuated by ND-13 treatment (p<0.05) (Fig 10B). Analysis of nigral dopaminergic cells immunoreactivity for tyrosine hydroxylase showed that ND-13 attenuated loss of TH-positive cells induced by MPTP exposure (Fig 10C–10E).

## Discussion

Effective, disease-modifying treatments are required for PD patients. DJ-1 could be a valuable target for exerting neuroprotection in PD, since it has protective abilities against endogenous and exogenous oxidative insults, largely by regulating the Nrf2 system [14–26, 35–38,39]. We designed a short DJ-1-derived peptide, comprising 13 amino-acids derived from DJ-1, conjugated to a 7 amino-acids CPP (named ND-13) with the aims of validating DJ-1 as a therapeutic target in PD and discovering a disease-modifying drug candidate,. In a series of experiments described above, ND-13 atttenuated significantly and robustly dopaminergic degeneration in experimental models of PD.

There is increasing evidence that the transcription factor Nrf2 is involved in the neurodegenerative process in PD. Under normal conditions, Nrf2 is bound to a kelch-like ECH-associated protein in the cytoplasm and degraded through the ubiquitin-proteasome pathway. Upon exposure to oxidative stress, it is released from kelch-like ECH-associated protein and translocates into the nucleus where it activates its target genes by binding to a conserved antioxidant response element [27, 28]. Activation of Nrf2 was shown to exert neuroprotection in experimental models of PD [40, 41]. Clements and colleagues [35] showed that DJ-1 increased the expression of Nrf2 target antioxidant genes by stabilizing the Nrf2 protein in a liver cell line and primary mouse embryonic fibroblasts. In mouse embryonic fibroblasts derived from DJ-1-knockout mice, the expression of Nrf2 target genes is suppressed [35]. The upregulation of NQO1, using the prototypical Nrf2 activator tert-butylhydroquinone (tBHQ), was repressed by DJ-1 siRNA [35]. Similarly, exposure of a human lung epithelial cell line to cigarette smoke, which causes oxidative modification of DJ-1 and enhances its proteasomal degradation, resulted in decreased Nrf2 stability and impaired antioxidant gene induction [38]. A recent study demonstrated that DJ-1 activated Nrf2 by increasing its protein levels, promoting its nuclear translocation and enhancing its binding to antioxidant response element sequence in Trx1 promoter, thereby increasing Trx1 levels [37]. These reports are consistent with our findings, demonstrating that ND-13, a DJ-1 derived peptide, activates Nrf2, upregulates antioxidant genes and exerts neuroprotection.

This manuscript describes a novel therapeutic strategy based on the DJ-1 protein. ND-13, a short peptide, the design of which is derived from the conserved sequence of DJ-1 and which is conjugated to a cell penetrating peptide and has been extensively evaluated in experimental models of PD. The peptide activated the Nrf2 system, reduced intracellular oxidative stress and resulted in increased tyrosine hydroxylase-positive dopaminergic cells in the substantia nigra and increased dopamine content in the barins of 6-hydroxydopamine and MPTP animal models of PD. However, we have to be aware of several limitations. Firstly, the PD models that we used are acute and the dopaminergic cells are damaged within hours or a few days while in patients these cells are in the process of degeneration for years. Secondly, the brain penetration and the pharmacokinetics of ND-13 have not yet been determined. Third, exposure to MPTP or 6-hydroxydopamine could induce loss of thyrosine hydroxylase immunoreactivity without cell death, since coincident with neuronal cell loss some nigral neurons lose TH expression [42] and this precedes dopaminergic cell death [43] in various toxic models. We have used well-established and researched protocols, yet direct neuronal cell death was not assessed in the *in vivo* models and theoretically ND-13 could preserve only dysfunctional dopaminergic cells and not reduce dopaminergic cell death. In vivo preservation of the dopaminergic system function was demonstrated by the behavioral assays, TH staining and brain dopamine content. In the *in vitro* studies ND-13 preserved cell viability of both neuronal cell line and primary cultured neurons exposed to oxidative stress, neurotoxins and trophic factors deprivation. Fourth, we have not detected any direct detoxifying effects of ND-13 on MPTP or 6-hydroxydopamine (data not shown). In the *in vitro* studies the peptide was thoroughly washed before toxic exposure. Yet, ND-13 could prevent dopaminergic toxicity partially by a direct effect on the toxic activity itself. To implement our findings in the clinic, we are obliged to analyze the stability of the peptide in the serum and in the brain, to explore the effective doses, to test the immunogenicity and the safety of the peptide.

In conclusion, our findings further reinforce the hypothesis that DJ-1 plays a key role in the cellular defensive response against oxidative stress and neurotoxins. These exciting results propose DJ-1 as a novel therapeutic target for exerting preventive effects in PD and ND-13, a DJ-1 derived peptide, as a drug candidate.
